# Lung nodule segmentation via semi-residual multi-resolution neural networks

**DOI:** 10.1515/biol-2022-0727

**Published:** 2023-10-24

**Authors:** Chenyang Wang, Wei Dai

**Affiliations:** Department of Electrical and Electronic Engineering, Imperial College London, London, UK

**Keywords:** deep neural networks, lung, semi-residual MCNN, cloud, IoT

## Abstract

The integration of deep neural networks and cloud computing has become increasingly prevalent within the domain of medical image processing, facilitated by the recent strides in neural network theory and the advent of the internet of things (IoTs). This juncture has led to the emergence of numerous image segmentation networks and innovative solutions that facilitate medical practitioners in diagnosing lung cancer. Within the contours of this study, we present an end-to-end neural network model, christened as the “semi-residual Multi-resolution Convolutional Neural Network” (semi-residual MCNN), devised to engender precise lung nodule segmentation maps within the milieu of cloud computing. Central to the architecture are three pivotal features, each coalescing to effectuate a notable enhancement in predictive accuracy: the incorporation of semi-residual building blocks, the deployment of group normalization techniques, and the orchestration of multi-resolution output heads. This innovative model is systematically subjected to rigorous training and testing regimes, using the LIDC-IDRI dataset – a widely embraced and accessible repository – comprising a diverse ensemble of 1,018 distinct lung CT images tailored to the realm of lung nodule segmentation.

## 1 Introduction

Lung disease constitutes a predominant and lamentable cause of mortality in both male and female populations [1]. Noteworthy among these ailments are lung cancer and respiratory infections like COVID-19, which collectively confront significant challenges in the realm of diagnostics. The prognosis of individuals grappling with pulmonary afflictions exhibits a discernible variance contingent upon the disease’s stage at the juncture of diagnosis. For instance, the prognosis for early-stage lung cancer treatment yields remarkably high survival rates. However, as the malaise advances, ushering in metastasis, the survival rates witness a precipitous decline, concomitant with the diminishing efficacy of therapeutic interventions. Regrettably, the symptoms of lung diseases frequently remain latent until an advanced disease stage has been reached. Furthermore, the symptomatic manifestations of lung cancer are frequently misconstrued, with patients often attributing them to long-term smoking consequences, thereby exacerbating diagnostic delays. It is against this backdrop that early screening for lung nodules emerges as a preeminent prophylactic strategy in the combat against lung cancer [2].

Lung nodules, minute external structures within the lung characterized by diameters spanning 3–30 mm, present a formidable challenge in detection due to their diminutive size. Their identification is compounded by their morphological resemblance to non-nodular structures, thus engendering ambiguity [3]. Traditional computer vision algorithms have historically grappled with the distinction between lung nodules and other pulmonary structures, necessitating an extensive reliance on the clinical acumen of radiologists for the early-stage diagnosis of lung cancer. The advent of deep learning, marked by recent advancements, has catalyzed the ascendancy of neural networks in the arenas of lung nodule detection, classification, and segmentation. It is imperative to underscore the pivotal nature of investigating image segmentation methodologies tailored explicitly for lung nodules. Given the inherent intricacies and intricacies of medical imaging, particularly underscored by the prevalent imbalances between nodule and non-nodule samples, the associated learning tasks and their application contexts impose rigorous demands upon the precision and interpretability of deep learning techniques.

Conventional techniques for image classification and detection predominantly yield generalized insights, encompassing aspects such as tumor location and typology. However, they regrettably fall short in furnishing the requisite granularity and precision sought in medical diagnoses. In stark contrast, segmentation methodologies have the potential to furnish pixel-level predictive maps, thereby endowing medical practitioners with the capacity to minutely scrutinize attributes such as precise location, shape, dimensions, and the numerical prevalence of potential lung nodules. Such pixel-level insights form a robust foundational bedrock for the identification and assessment of risk and malignancy associated with each distinct pulmonary tumor.

A pioneering milestone in harnessing neural networks for image segmentation was achieved through the advent of Fully Convolutional Networks (FCN) [4]. Within this paradigm, the neural network is imbued with the transformative capacity to process the original image. The process begins with the computation of a feature map, which is subtly scaled in size through a sequence of convolutional operations and sequential downsampling layers. This preliminary computation engenders a critical substrate. Subsequently, the network embarks on the discrete prediction of classification outcomes for each individual pixel nestled within the feature map. This orchestration culminates in the synthesis of a high-level heatmap, emblematic of the synthesized spatial organization of the target entities.

Expanding upon the bedrock established by FCN, the U-Net architecture introduces a novel interpretative layer, effecting a pivotal augmentation [5]. Herein, an expansive decoder pathway is ingeniously woven into the framework. The purpose of this decoder trajectory is to accurately remap the downsampled feature map, resulting in the generation of an improved heatmap. This heatmap, hewn from the encoded dimensions, duly proffers a segmentation map – a visual artifact that faithfully preserves the resolution of the source image. Functionally, the encoder trajectory of U-Net embraces the mantle of feature extraction, nurturing the discernment of salient attributes. Conversely, the decoder pathway is tasked with the precise localization of feature points, culminating in a masterful restoration of the original spatial resolution. Notably, this duality of functional trajectories imparts a symmetrical disposition, coalescing into a silhouette that evocatively mirrors a “U” shape.

U-Net’s efficacy in diverse image segmentation scenarios has yielded a progeny of variants. Notable among these are Residual U-Net, U-Net++, Bi-Directional ConvLSTM U-Net (BCDU-Net), and Attention U-Net [6,7,8,9]. Residual U-Net innovatively incorporates residual connections to mitigate network degradation. U-Net++, a departure from the norm, redesigns skip pathways to enhance model robustness through cross-convolutional information fusion. BCDU-Net introduces LSTM modules to foster efficient feature propagation, enhancing the coupling of encoder and decoder outputs. Attention U-Net employs an attention mechanism to refine focus on salient regions, suppressing inconsequential backgrounds. These U-Net variants optimize distinct components, consistently yielding improved segmentation outcomes across varied image segmentation tasks.

Efforts to cross-pollinate from disparate deep learning domains into image segmentation have yielded noteworthy results. Mask R-CNN, a consequential two-stage object segmentation network, extends Faster R-CNN via a novel branch dedicated to target mask prediction [10,11]. This technique finds utility in scenarios where computational resources are sufficient for tackling minuscule targets. UNETR leverages the Vision Transformer concept within the U-Net framework, harnessing multiple transformers in the encoder to augment global context and spatial dependency learning [12]. However, despite their triumphs, a salient limitation resides in their reliance on single final outputs for model training. Deeper layer feature outputs remain underutilized, potentially impacting convergence efficiency and ultimate training outcomes.

In the year 2020, a comprehensive neural architecture denominated as the multi-resolution convolutional neural network (MCNN) surfaced, uniquely tailored to address diverse image-to-image inverse predicaments [13]. The fundamental tenet underpinning MCNN resides in its ingenious incorporation of multiple branches within concealed strata of the neural network structure, thereby engendering predictions at varying resolutions. This pioneering design philosophy has found extensive validation across a gamut of computational challenges, spanning domains such as image super-resolution, denoising, and phase retrieval. Empirical evaluation has evidenced the substantial augmentation in model training stability, coupled with the triumphant outperformance vis-à-vis an array of state-of-the-art methodologies across each respective domain.

While MCNN inherently bears semblance to the U-Net paradigm, a discernible departure is underscored by MCNN’s augmentation via the integration of multiple branches into the decoder architecture [13]. These branches intertwine with supplementary convolutional layers, thereby furnishing auxiliary outputs encapsulating distinct resolutions. The crux of the loss function transpires in the comprehensive juxtaposition of outputs and annotation maps spanning the spectrum of resolutions, a departure from the conventional approach of isolating the final output solely at the original resolution.

It warrants acknowledgment that although MCNN’s original intent did not encompass image segmentation undertakings, the striking parallels it shares with U-Net tantalizingly beckon its potential application in the realm of lung nodule segmentation. The inherent architectural blueprint of MCNN conveys an inherent propensity to acquire insights from diverse resolutions, manifesting as an asset particularly germane to tasks such as lung nodule segmentation. In the context of datasets characterized by a pronounced imbalance between positive and negative samples, the adoption of MCNN’s architectural paradigm emerges as a strategic overture, catalyzing swift and incisive approximations of nodule localization at a macroscopic level. Subsequent network strata delve deeper into the finer intricacies of nodule boundaries at a pixel-level, culminating in a cascading refinement.

Notably, the incorporation of multi-resolution branches within the U-Net architecture mandates only a marginal augmentation of computational resources [13]. This conveys the salient advantage of imperceptibly infusing the utilization of multi-resolution output heads without obfuscating the original usage scenarios of the U-Net paradigm.

Presently, the broader landscape of scholarship has largely remained reticent concerning the potential integration of MCNN into the ambit of lung nodule segmentation [13]. In response, this research endeavor aspires to bridge this void, contemplating the viability of this application. Drawing inspiration from a medley of U-Net variants, this inquiry sets forth the ambitious objective of conceiving a model solution that bears the hallmark of reusability. The ultimate ambition is succinctly encapsulated within the twin imperatives of heightening the precision and efficiency of automated lung nodule segmentation across authentic, real-world datasets.

## 2 Methodology and model structure

This section expounds upon the comprehensive procedure employed for the construction of the lung nodule segmentation model through the innovative semi-residual MCNN method. The endeavor unfolds through distinct phases, meticulously delineated within the ensuing subsections.

### 2.1 Dataset and pre-processing

Adherence to the pertinent regulations underscores every facet of the experimental endeavors undertaken in this project. The foundational cornerstone of this investigation resides within the Lung Image Database Consortium and Image Database Resource Initiative (LIDC-IDRI) Dataset, an open repository generously endowed with the prerogative of browsing, downloading, and deployment for commercial, scientific, and pedagogical pursuits, all under the umbrella of the Creative Commons Attribution 3.0 Unported License. Gratitude is extended to the National Cancer Institute and the National Institutes of Health Foundation, whose collaborative contributions have engendered this freely accessible database [14].

The LIDC-IDRI dataset contains 1,018 CT images from 1,010 patients, annotated by 4 experienced chest radiologists. The data in this dataset were obtained from seven different academic institutions, using different scanners and their parameters, thus ensuring a wide distribution of data. In this project, we examined nodules that were annotated by all four radiologists and were at least 3 mm in diameter, following previous studies [15,16]. To construct each CT scan’s ground truth segmentation map, a 50% consensus criteria was applied for each annotated nodule.

The LIDC-IDRI dataset was chosen to train and evaluate the model for this experiment because it has a large amount of data and high-quality annotations. Among 1,018 CT images in this dataset, 200 images between LIDC-IDRI-0730 and LIDC-IDRI-0951 were randomly selected as the test set and 540 images from the remaining dataset were randomly selected as the training and validation sets. These images are randomly shuffled, and 390 of them are assigned to the training set, while the remaining 150 images are assigned to the validation set. The data splitting approach aims to distinguish the data distribution of the test set data from that of the training and validation sets, thus better reflecting the model’s ability to predict random data samples in the real world.

All raw CT images have the size of 512 × 512 × *Z*, where *Z* varies as the slice thickness of each CT image changes. However, the designed input size of this model is 256 × 256 × 32, and it is essential to resize and crop the original image to fit the model. The data pre-processing procedures are as follows:

(1) Reduce the image to half its original size in *X*-*Y* directions. The resized image has a size of 256 × 256 × *Z*.
(2) Iterate over every slice of the image and compare it with its corresponding annotation map. If the slice contains a nodule sample, keep the slice in the image. Otherwise, discard this slice. The shape of the image becomes 256 × 256 × *Z*′.
(3) If *Z*′ is smaller than 32, take additional subsequent non-nodule slices to make complete volume data.
(4) Randomly sample one or several 256 × 256 × 32 image blocks from the processed image. Add these sampled images to the training or validation set.

**Figure 1:**
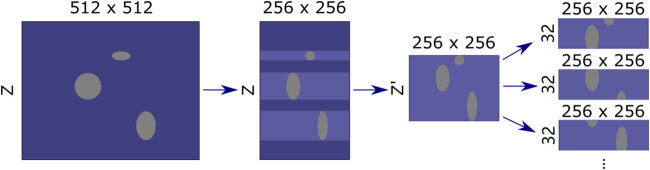
Overview of image crop and resize methods. The horizontal direction corresponds to the *x* and *y* axes and the vertical direction corresponds to the *z* axis. Purple color indicates non-nodule regions and gray color indicates nodule regions.

An overview of the aforementioned methods is depicted in Figure 1. In comparison to conventional techniques that solely extract data from the vicinity of nodules, the method of this experiment aims to preserve extensive global information. This encourages the model to undertake not only the fundamental segmentation task but also to identify nodule targets that might be merely tens of pixels in size, originating from the original image that encompasses millions of pixels. This situation presents a challenge to the model’s capacity for detecting small targets.

Furthermore, by exclusively retaining the data layers containing at least one nodule pixel, the density of positive samples is enhanced without compromising the global information. This, in turn, mitigates the issue of substantial imbalance between positive and negative samples and reduces the complexity associated with model training.

In a raw CT image, the pixel values are quantified using the Hounsfield Unit (HU), a measurement of radiation absorption and attenuation within tissues [17]. Following established research conventions, those pixels with HU values ranging from –1000 to 600 were solely retained. This filtration process eliminates elements like water, air, and body tissues that lack relevance to the current project. Subsequently, the filtered region is normalized to a range of 0–1 through the utilization of the following function:

(1) \[{I}_{\text{normali}\text{z}\text{ed}}=\frac{I-{I}_{\min }}{{I}_{\max }-{I}_{\min }},]\]

where \[{I}_{\max }=1,000\text{and}{I}_{\min }=-600]\] .

**Figure 2:**
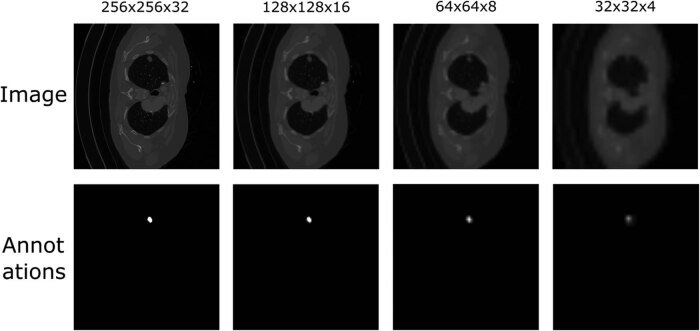
An example of multi-resolution image and annotations to train on MCNN. Only one slice of 3-dimensional image and annotation is shown per resolution scale.

To enable the network’s training with multi-resolution outputs, low-resolution annotations are generated through an iterative resizing procedure applied to the original annotated segmentation map, utilizing a reduction factor of 0.5. Figure 2 provides an illustrative depiction of the resulting pre-processed data across various resolution scales.

Data augmentation techniques, such as geometric and pixel transformations applied to raw data, have been proven effective in augmenting data volume and preventing overfitting. Nonetheless, in the context of our dataset, these methods do not yield substantial benefits. The inherent complexity of the original data and the potential for these techniques to introduce noise to training samples typically result in suboptimal training outcomes. Therefore, only random image flips were used on either *x*-axis or *y*-axis.

### 2.2 Training device

Training neural networks on three-dimensional images demands substantial computing power, and the utilization of a capable GPU significantly enhances the efficiency of network computations. For this project, the execution took place on an online server equipped with 6 Intel^®^ Xeon^®^ Gold 6142 CPU and an NVIDIA^®^ RTX 3090 graphics card featuring 25.4 GB of graphic memory.

### 2.3 Model structure

Every blue box in the visualization represents a multi-channel feature map, with the number of channels indicated at the top of each box. The dimensions in the *x*-*y*-*z* axes are presented on the lower left edge of the initial box within each resolution. Gray boxes signify duplicated feature maps, while arrows delineate distinct operations.

**Figure 3:**
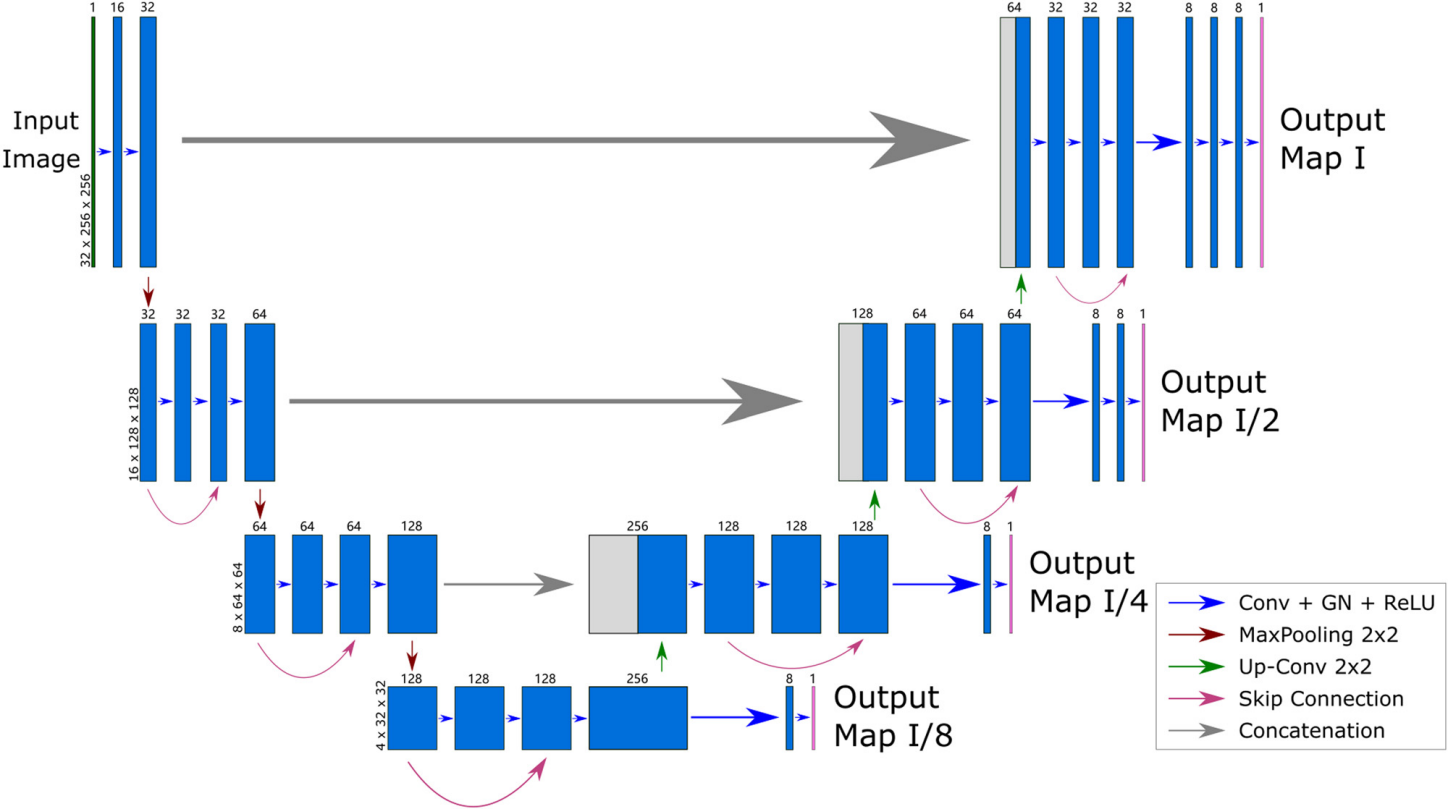
Overview of semi-residual MCNN architecture.

In this project, a custom architecture named the semi-residual MCNN, visually represented in Figure 3, was devised. The architecture encompasses both a contracting and an expansive path. The contracting path functions as an encoder, extracting features from the original image. On the other hand, the expansive path serves as a decoder, mapping these derived features back to the original image and generating output segmentation maps across various resolution scales. The core components of the model are semi-residual building blocks.

Each semi-residual building block comprises three consecutive 3 × 3 convolutions, each accompanied by a Group normalization (GN) layer and a ReLU activation. Notably, a skip connection path is introduced between alternate convolutional layers within each block. The initial building block in the contracting path is somewhat simplified for enhanced computational efficiency. Moreover, within the contracting path, a 2 × 2 max-pooling operation is inserted between neighboring semi-residual building blocks at each step. Conversely, the expansive path incorporates a 2 × 2 up-convolutional layer in every step, halving the channel count, and involves concatenation with the corresponding feature map from the contracting path.

Crucially, the expansive path links to four output heads through the feature map. Each of these output heads encompasses one or more 3 × 3 Convolution + GN + ReLU intermediate layers, culminating in an output convolutional layer. Ultimately, these output heads generate segmentation maps at distinct resolution scales.

### 2.4 Loss and metrics

Drawing inspiration from other cutting-edge image segmentation networks, the classic binary cross-entropy loss (BCEloss) have been adopted to train networks [18]. In essence, binary cross-entropy evaluates each predicted probability against the corresponding actual class outcome, which can assume values of either 0 or 1. It subsequently calculates a score that penalizes probabilities according to their deviation from the predicted value. The mathematical expression for BCEloss is as follows:

(2) \[\text{BCEloss}=-\frac{1}{N}\mathop{\sum }\limits_{i=1}^{N}{y}_{i}\log (p({y}_{i}))+(1-{y}_{i})\log (1-p({y}_{i})),]\]

where *y*_*i*_indicates the real value of each pixel and *p*(*y*_*i*_) indicates its predicted value.

In terms of evaluation metrics, the dice coefficient is used as the main metric to evaluate the quality of model prediction outcomes. The dice coefficient, also known as the Sørensen–Dice index, is a statistical tool that evaluates the similarity between two sets of data [19]. The General formula of the dice coefficient is as follows:

(3) \[\text{Dice}=\frac{2\times \text{True\_Positives}}{2\times \text{True\_Positives}+\text{False\_Positives}+\text{False\_Negatives}}.]\]

The dice coefficient strikes a balance between false negative and false positive samples, contributing to an overall measure of similarity. This attribute makes it particularly adept at evaluating lung nodule segmentation results, as it disregards true negative samples. In this experiment, the dice coefficient is employed to assess the prediction outcomes of both the validation and test sets, providing insights into prediction quality for both scenarios.

Furthermore, precision and recall are incorporated as supplementary metrics, offering assessments of a model’s direct costs and opportunity costs, respectively. The formulas for these metrics are as follows:

(4) \[\text{Precision}=\frac{\text{True\_Positives}}{\text{True\_Positives}+\text{False\_Positives}},]\]

(5) \[\text{Recall}=\frac{\text{True\_Positives}}{\text{True\_Positives}+\text{False\_Negatives}}.]\]

### 2.5 Optimization techniques

The learning rate stands out as a pivotal hyper-parameter in the training process, exerting a profound impact on the training outcome. Its selection warrants meticulous consideration. An effective approach to learning rate management involves initiating with a higher value to swiftly navigate the vicinity of a minimum region. Subsequently, a gradual decay towards a smaller value aid in pinpointing the local minimum point. This strategy contributes to optimizing the model’s convergence and overall performance.

Concerning this strategy, the step decay method was chosen as the learning rate scheduler in this project, with an initial learning rate equal to 2 × 10^−4^ and decay by a factor of 0.6 every 25 epochs [20]. According to experimental result, the network converges as the training process continues, where the learning rate gradually drops to 7.25 × 10^−7^.

In terms of the optimizer, Adam was chosen in this project with the default exponential decay rate factors of *β*_1_ = 0.9 and *β*_2_ = 0.999. Compared with another commonly used optimizer, SGD with momentum, Adam generally enables the model to converge faster and leads to better prediction results [16,21].

## 3 Results and analysis

### 3.1 Results

Data distribution in the test set is described in Table 1. Out of 200 images in the test set, 103 contain at least one nodule pixel, while the remaining 97 images contain no nodule pixels at all. All test set images with at least one positive pixel are split into three categories based on the density of nodule pixels. To be more specific, images with a ratio between nodule and non-nodule pixels smaller than 5 × 10^−6^ fall in the category SPARSE, images with a ratio between 5 × 10^−6^ and 2 × 10^−5^ falls into the category MODERATE, and those with a ratio greater than 2 × 10^−5^ falls in the category DENSE. The amount of data that fall in each category is shown in Table 1.

**Table 1:** Data distribution in the test set

Total images	SPARSE	MODERATE	DENSE	Fully negative
200	34	31	38	97

In this study, seven models were set up to compare performance with our method and other state-of-the-art approaches. These models are basic MCNN, residual MCNN, semi-residual U-Net, semi-residual MCNN (the solution of this experiment), semi-residual MCNN with squeeze-and-excitation modules, semi-residual MCNN without GN, and 3D Recurrent DenseUNet (proposed in 2020, achieved state-of-the-art performance on another similar lung nodule segmentation dataset named NSCLC) [22].

Dice coefficients, precision, and recall are evaluated on the test set with at least one nodule pixel, while the percentage of correct prediction and average false positive pixels per image are evaluated on the test set with non-nodule pixels only.

**Figure 4:**
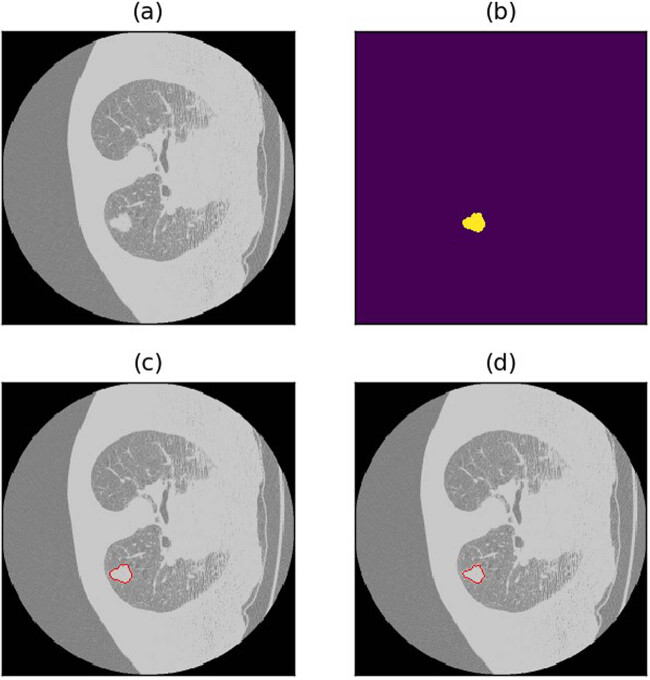
Result visualization of patient LIDC_IDRI_0785. (a) Raw image. (b) Annotations. (c) Annotated image. (d) Raw image annotated with predicted result.

Tables 2 and 3 summarize the lung nodule segmentation performance of each method, and Figure 4 shows a visualized example of a good prediction result using semi-residual MCNN.

**Table 2:** Results on the test set with at least one positive pixel

Model type	Dice_sparse	Dice_moderate	Dice_dense	Dice_average	Precision	Recall
Basic MCNN	0.2302	0.4824	0.5942	0.4380	0.8091	0.4277
Residual MCNN	0.2250	0.4736	0.5248	0.4080	0.7550	0.3781
Semi-residual U-Net	0.1692	0.4258	0.5001	0.3660	0.7610	0.3618
Semi-ResMCNN (SE)	0.1578	0.3672	0.5274	0.3397	0.8845	0.3055
Semi-ResMCNN (NO GN)	0.0378	0.0984	0.2750	0.1436	N/A	N/A
Semi-residual MCNN	0.2484	0.4767	0.6011	**0.4479**	0.7962	0.4391
Recurrent DenseNet	0.2800	0.4627	0.5437	0.4204	N/A	N/A

**Table 3:** Results on the test set with no positive pixels

Model type	Correct rate	Average FP per image
Basic MCNN	0.6907	51.59
Residual MCNN	0.6701	31.30
Semi-residual U-Net	0.6598	39.41
Semi-residual MCNN (SE)	0.7528	13.60
Semi-residual MCNN (NO GN)	0.4226	246.7
Semi-residual MCNN	0.6701	36.68
Recurrent DenseNet	0.3505	N/A

According to the results above, semi-residual MCNN achieved average dice coefficients of 0.4479, which is the highest among methods. A few significant observations are as follows:

(1) The incorporation of GN and multi-resolution output head modules yields a substantial enhancement in the comprehensive performance of network.
(2) Out of the basic MCNN, semi-residual MCNN, and residual MCNN architectures, it is evident that the semi-residual MCNN achieves the most superior performance. This outcome underscores the advantageous impact of employing semi-residual building blocks in achieving enhanced performance.
(3) In nearly all network configurations, a notable trend emerged wherein precision attained exceptionally high values, while recall remained relatively low. This observation indicates that instances of missed detection are significantly more probable than instances of misdetection across all cases.
(4) In scenarios involving images devoid of positive pixels, the semi-residual MCNN exhibits superior performance compared to Semi-ResMCNN (NO GN) and Recurrent DenseNet. However, its performance remains comparable to that of other employed methods.

Broadly, the superiority of semi-residual MCNN over other methods can be attributed to three pivotal components: the semi-residual building block, GN, and multi-resolution output heads. A comprehensive breakdown of each of these components is provided in the subsequent sections for detailed analysis.

### 3.2 Component analysis

#### 3.2.1 Semi-residual U-Net building block

Earlier studies have highlighted that the incorporation of residual connections can alleviate the problem of network degradation [23]. Nevertheless, based on experimental findings, it remains beneficial to retain certain basic convolutional units within the network. This ensures that feature maps are comprehensively utilized before advancing to subsequent layers. In light of this consideration, we introduce semi-residual building blocks to network. These building blocks are structured by integrating residual connections that bypass two convolutional units, while maintaining one unskippable unit within each building block. This strategic arrangement effectively balances the utilization of feature maps while harnessing the advantages of residual connections.

**Figure 5:**
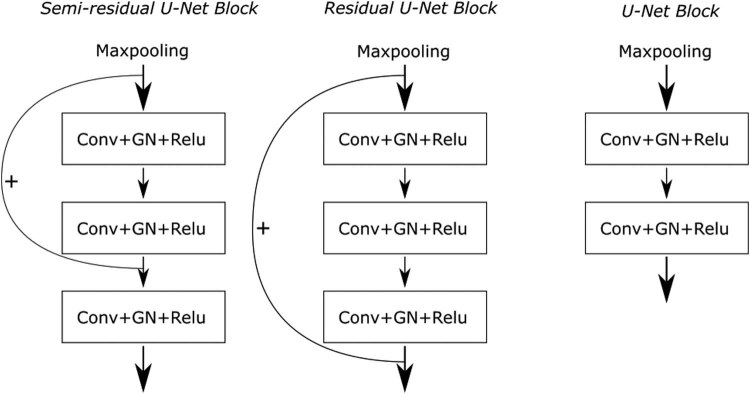
Comparison between the structure of semi-residual U-Net block, residual U-Net block, and U-Net block in the encoder section.

**Figure 6:**
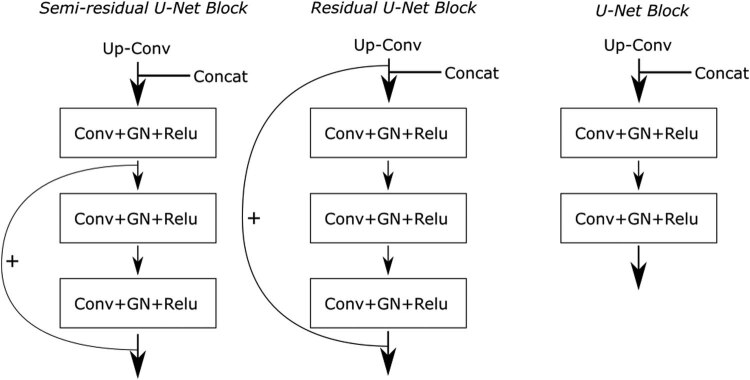
Comparison between the structure of semi-residual U-Net block, residual U-Net block, and U-Net block in the decoder section.

Figures 5 and 6 provide a comparative analysis of the internal structures of various types of building blocks. The key distinction between a semi-residual U-Net block and a residual U-Net block lies in the residual connection setup. In the case of a semi-residual U-Net block, the residual connection skips over 2 out of the 3 convolutional units within the block. Conversely, a residual U-Net block employs a residual connection that bypasses all convolutional units. Employing semi-residual U-Net blocks offers a significant advantage. Under extreme conditions where parameters of convolutional layers along the skip connection path become zero, a standard convolutional operation remains operative for data processing at every resolution scale. This strategic design ensures that essential feature extraction operations are consistently performed while leveraging residual connections to avert network degradation. As a result, a more balanced training approach is achieved compared to residual U-Net blocks. It is worth noting that a U-Net block, distinct from the aforementioned designs, lacks residual connections and incorporates a shallower structure. This mitigates network degradation concerns while maintaining effective performance.

Table 4 presents the outcomes of experiments on the training, validation, and test sets using models constructed with U-Net blocks, residual U-Net blocks, and semi-residual U-Net blocks, respectively. Notably, several key observations emerge from these results. First, the model employing U-Net building blocks exhibits the least favorable performance on the training set, marked by the lowest training loss and training dice coefficient among the three networks. This suggests that the U-Net block model is encountering underfitting issues, failing to adequately capture the complexity of the training data. On the other hand, the model integrating semi-residual building blocks demonstrates marginally improved outcomes, not only on the training set but also across the validation and test sets compared to the U-Net block model. This indicates that the semi-residual U-Net block model has a better ability to generalize beyond the training data, avoiding significant underfitting concerns. Interestingly, the residual U-Net block model showcases the most effective fit to the training set, evident from superior training outcomes. However, this strength translates to poorer results in terms of validation loss and test dice coefficient. This discrepancy indicates that the residual U-Net block model is grappling with overfitting, as it exhibits excessive adaptation to the training data at the expense of generalization. In summation, these findings underscore the advantages of the semi-residual U-Net block model, which achieves a balance between fitting the training data and generalizing to new, unseen data.

**Table 4:** Optimal results comparison between models built with U-Net blocks, residual U-Net blocks, and semi-residual U-Net blocks over 300 epochs

Block type	train_loss	train_dice	val_loss	val_dice	test_dice
U-Net	1.55 × 10^−5^	0.6539	5.97 × 10^−5^	0.4515	0.4380
Residual U-Net	1.17 × 10^−5^	0.6722	8.50 × 10^−5^	0.4622	0.4080
Semi Residual U-Net	1.50 × 10^−5^	0.669	4.19 × 10^−5^	0.4553	0.4479

#### 3.2.2 GN

Batch normalization (BN) is a common and useful method in training networks to improve training stability and efficiency [24]. However, one disadvantage of using BN is that when the batch size is small, the standard deviation is likely to be very tiny within the mini-batch, resulting in numerical instability in normalization. This issue becomes evident in 3D image segmentation tasks, as a substantial amount of memory is required to store high-dimensional training data. In practice, the maximum batch size that NVIDIA RTX 3090 GPU could accommodate is only 2, which is very likely to result in numerical instability during training.

Layer normalization (LN) is an alternative normalization method that bypasses the issue of batch size [25]. Instead of BN normalizing each mini-batch, LN normalizes each data across all channels for every single image in a mini-batch. In this case, all spatial information for each image is preserved. Most of the extracted key features are retained in the normalization process and lead to better training outcomes than BN.

However, normalizing the whole input layer could be computationally expensive for three-dimensional inputs. Thus, GN was proposed as a generalized method of LN by selecting a few groups for each normalization to be computed along with the layer [26]. It is worth mentioning that when there is only one group, GN becomes LN. When the number of groups equals the number of channels, GN becomes instance normalization (IN), where information between channels is not considered [27]. In general, GN leads to better training results and improved computational efficiency compared to LN and IN. A good explanation is that GN allows different distributions to be learned for each group of channels, which is a regularization method to improve the model’s generalization capability.

Illustrated in both Figures 5 and 6 within the preceding subsection, GN is implemented following each convolutional operation. The group size for each GN layer has been chosen as a hyperparameter to fine-tune during training endeavors. Notably, both group sizes of 4 and 8 have resulted in optimal prediction outcomes for the model. A comparison of experimental results is detailed in Table 5.

**Table 5:** Optimal result comparison between models built with GN and without GN

Model type	Train loss	Train dice	Val loss	Val dice	Test dice
Without GN	1.64 × 10^−5^	0.6331	3.11 × 10^−4^	0.3785	0.1436
With GN	1.50 × 10^−5^	0.669	4.19 × 10^−5^	0.4553	0.4479

**Figure 7:**
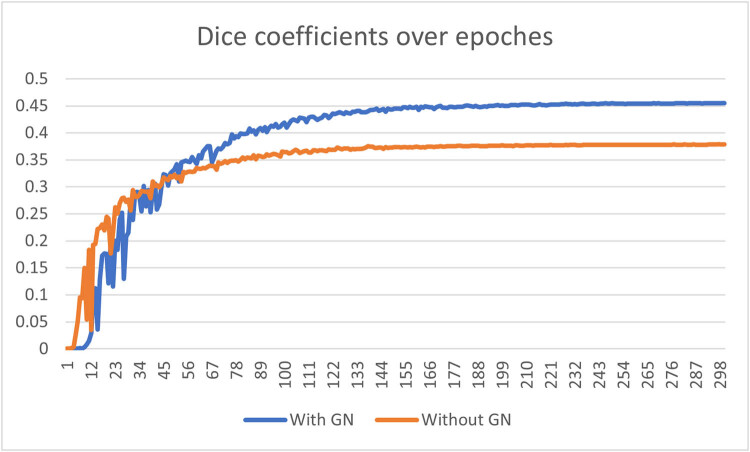
Comparison of dice coefficient on the validation set over epochs between the semi-residual MCNN with GN and the semi-residual MCNN without GN.

It is evident that GN plays a pivotal role in two crucial aspects: enhancing the model’s convergence on the training set and effectively regularizing the model to achieve a superior fit on both the validation and test sets. In terms of dice coefficients on the validation set, Figure 7 presents a comparison of the curves over 300 epochs between a model without GN and one with GN during the training phase. It is evident that the model without GN encounters challenges in optimization after 100 epochs, whereas the model with GN exhibits steady convergence and ultimately outperforms the GN-absent counterpart significantly.

Furthermore, when the training was conducted using BN, a distinctive pattern was noted, i.e., while the training loss and dice coefficient converge, the validation loss escalates substantially, and the validation dice coefficient nears zero. This observation serves as confirmation that BN is unsuitable as a normalization method in scenarios where the batch size is minimal.

#### 3.2.3 Multi-resolution output heads

As shown in the structure overview of semi-residual MCNN in Figure 3, four multi-resolution output heads are added to the generated feature maps from the decoder at each resolution scale. For each multiresolution head, several convolutional layers are introduced before the final output convolutional layer for smoothing and refinement purposes. The resolution of these output segmentation maps is 4 × 32 × 32, 8 × 64 × 64, 16 × 128 × 128, and 32 × 256 × 256, respectively. Only the head with outputs having the same resolution as the input images is considered a main output path, while the remaining three low-resolution heads are considered auxiliary output paths.

The following formula computes the training loss:

\[{L}_{\text{train}}={L}_{\text{main}}+\alpha ({L}_{\text{aux}\_1}\text{}+{L}_{\text{aux}\_2}+{L}_{\text{aux}\_3})]\]

where *L*_mean_ is the BCEloss of the main output, *L*_aux_*n*_is the BCEloss of the *n*th auxiliary output, and *α* is a coefficient starting from 0.4, decaying by a factor of 0.8 every 25 epochs.

The rationale behind introducing a penalty to the auxiliary loss stems from the fundamental principle that the main output remains the primary task, whereas auxiliary outputs are auxiliary in nature, aiding the model in grasping low-level features. These auxiliary outputs do not take precedence. As the model progressively converges, this factor should gradually decrease, empowering the model to center its attention on refining based on the ultimate output. Notably, during the validation and testing phases, auxiliary outputs are disregarded. Consequently, the loss function for the network in these stages effectively reduces to the BCELoss solely for the main output.

**Figure 8:**
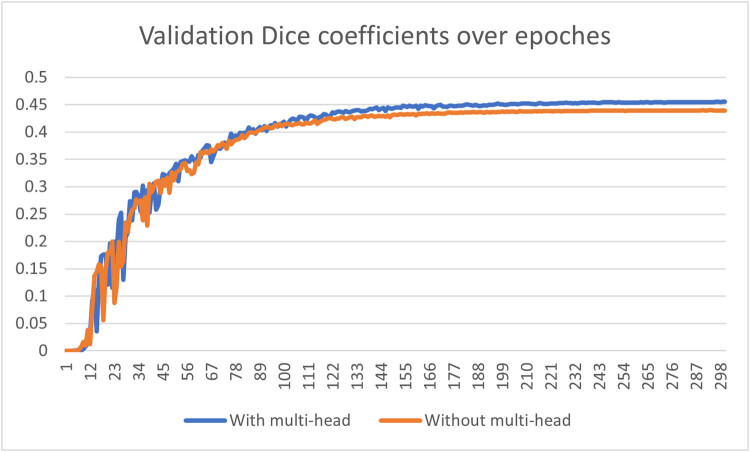
Comparison of dice coefficient of the validation set over epochs between the model with multi-resolution output heads and the model without multi-resolution output heads.

As illustrated in Figure 8 and corroborated by Table 6, the utilization of multi-resolution output heads has yielded a substantial enhancement in model performance across nearly all metrics. Focusing on the dice coefficient curve, both the model featuring multi-resolution outputs and the model devoid of such outputs exhibit comparable validation outcomes during the initial 100 epochs. However, subsequent to this point, the model lacking multi-resolution output heads encounters challenges in achieving convergence. In contrast, the model incorporating multi-resolution output heads demonstrates a gradual yet consistent convergence, ultimately attaining a superior final outcome.

**Table 6:** Optimal result comparison between models built with multi-resolution output heads and without multi-resolution output heads over 300 epochs

Model type	train_loss	train_dice	val_loss	val_dice	test_dice
Without multi-head	1.91 × 10^−5^	0.6377	4.13 × 10^−5^	0.4398	0.3660
With multi-head	1.50 × 10^−5^	0.669	4.19 × 10^−5^	0.4553	0.4479

Turning attention to the model’s performance on the test set, the incorporation of multi-resolution output heads has yielded a noteworthy improvement. The dice coefficient on the test set has escalated from 0.3660 to 0.4479, marking a substantial increase of 22.4%. This reinforcement highlights the tangible advantage of integrating multi-resolution output heads in elevating the model’s efficacy.

The rationale behind the efficacy of auxiliary outputs lies in their ability to activate hidden layers and facilitate improved gradient propagation within the networks. When a deep neural network is trained without auxiliary outputs, the optimization and evaluation of parameters within the network’s low-level features rely solely on other higher-level network layers. This process can potentially trigger network degradation and gradient vanishing issues due to the intricate and lengthy sequence of computations involved.

Furthermore, this approach can lead to a situation where the outputs of low-level hidden layers become inconsequential to the primary task. In such cases, since all parameters in the hidden layers exclusively contribute to the final outcomes, the low-level convolutional layers may struggle to extract pertinent features that directly align with the ultimate objective without direct supervision.

The integration of auxiliary outputs serves to alleviate these two concerns. By introducing auxiliary outputs, low-level features attain meaningful significance, and the pathways between hidden layers and output layers are truncated [28]. This configuration not only imparts significance to low-level features but also shortens the paths between different layers, mitigating the issues of network degradation, gradient vanishing, and the diminishing relevance of low-level hidden layers in the network’s learning process.

In the context of MCNN within this specific scenario, the overarching objective of employing a decoder lies in effectively pinpointing and localizing the feature information that has been extracted by the encoder. The integration of auxiliary output paths further serves to guide the model’s attention towards learning parameters that yield enhanced outcomes at each distinct resolution scale.

As the network delves deeper into its architecture, the model’s emphasis gradually shifts towards fine-tuning prediction boundaries and intricate features. This approach holds the potential to deter unnecessary pixel-level sprawl and, in turn, amplifies the efficiency of parameter utilization. The interplay between encoder and decoder, along with the introduction of auxiliary outputs, facilitates a more refined and strategic learning process, ultimately contributing to heightened model performance and more effective utilization of parameters.

### 3.3 Bad case analysis

**Figure 9:**
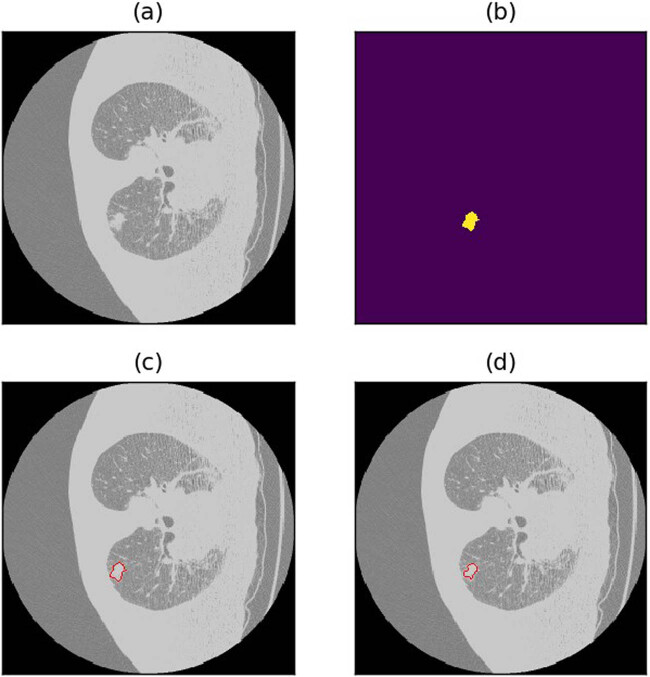
Example of shrunk borders. (a) Raw image. (b) Annotations (c) Annotated image. (d) Predicted image.

**Figure 10:**
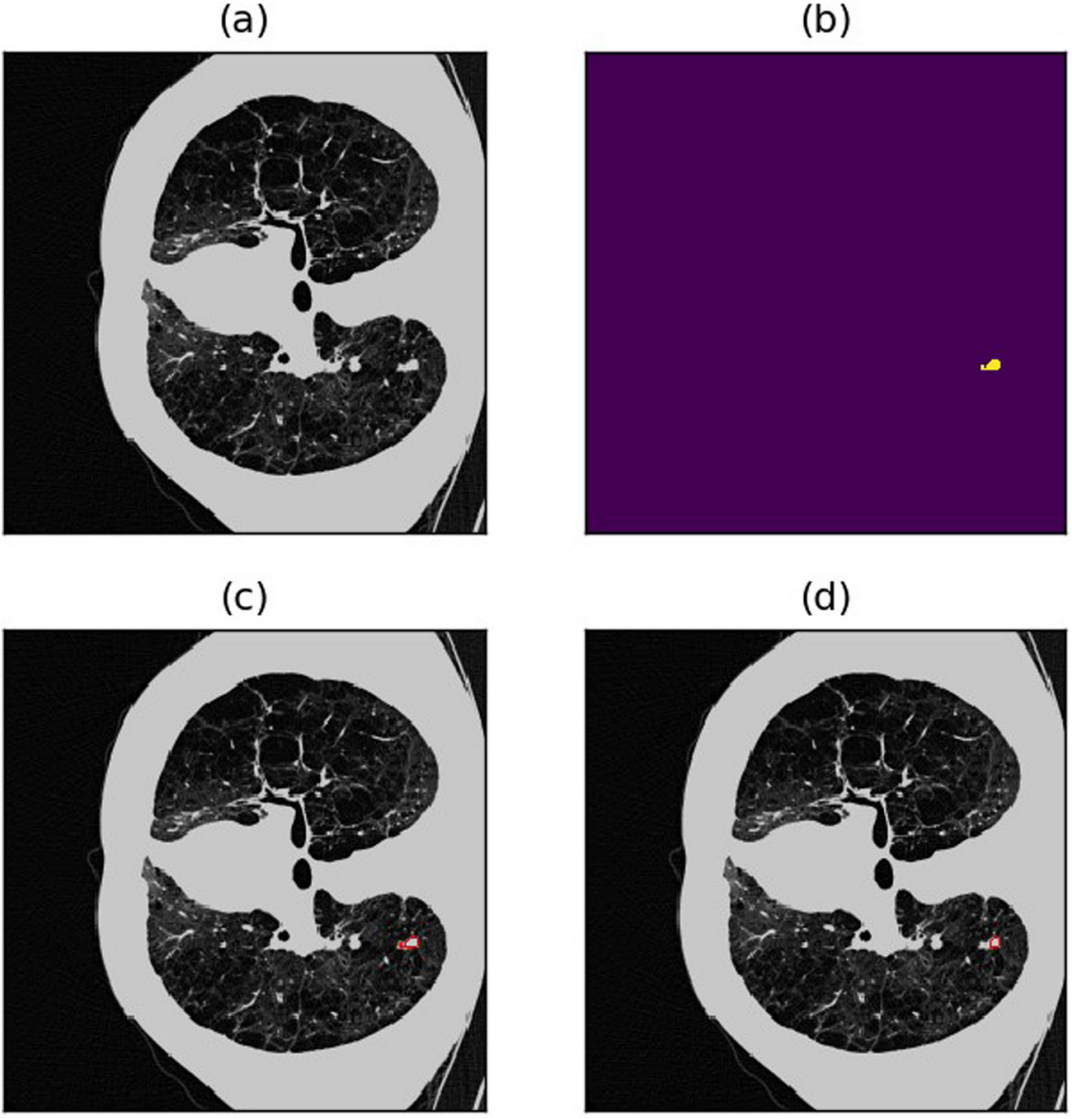
Example of shrunk borders. (a) Raw image. (b) Annotations (c) Annotated image. (d) Predicted image.

**Figure 11:**
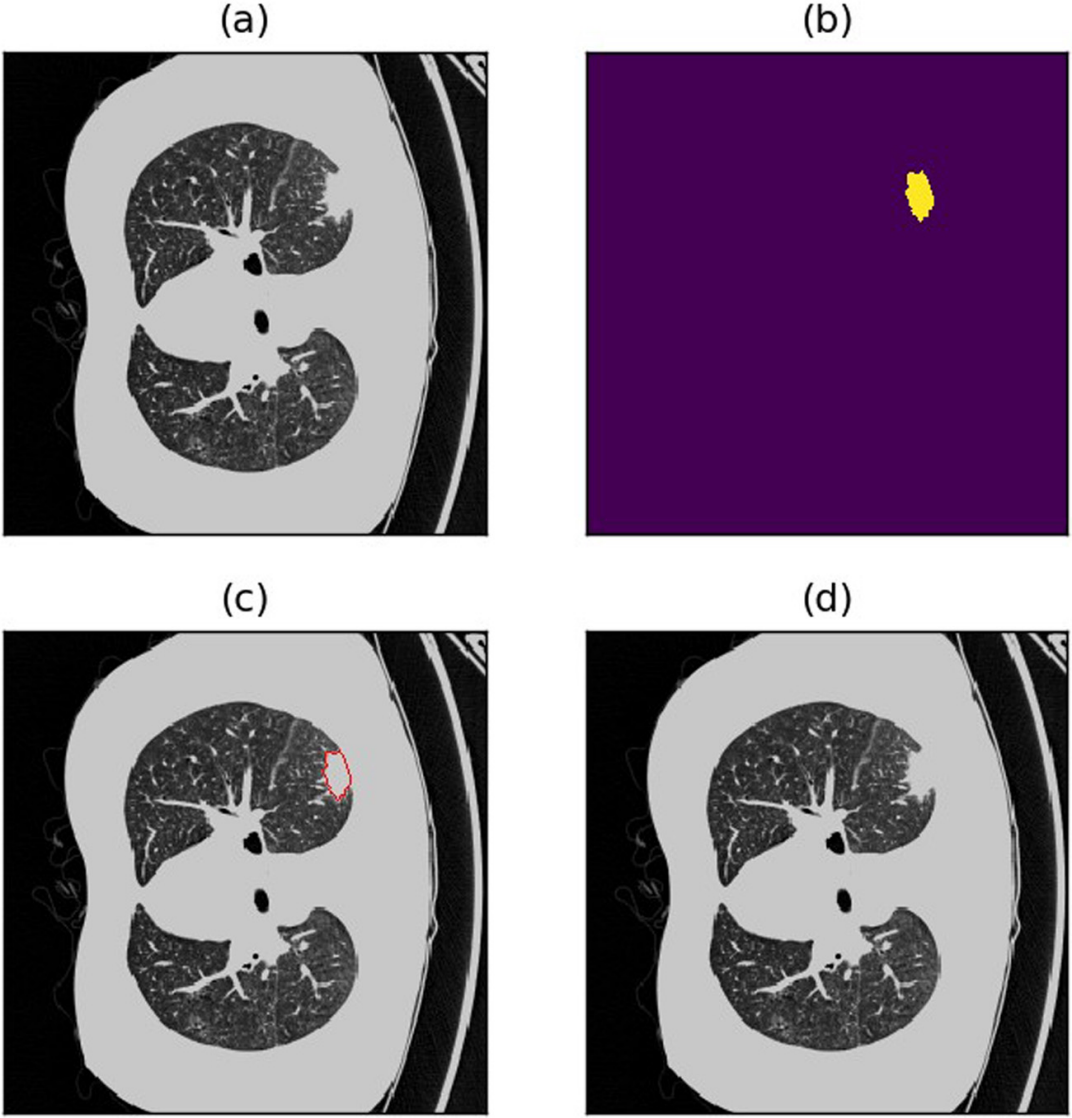
Example of missed detection due to lung nodules too close to the lung margins or other lung tissues. (a) Raw image. (b) Annotations (c) Annotated image. (d) Predicted image.

**Figure 12:**
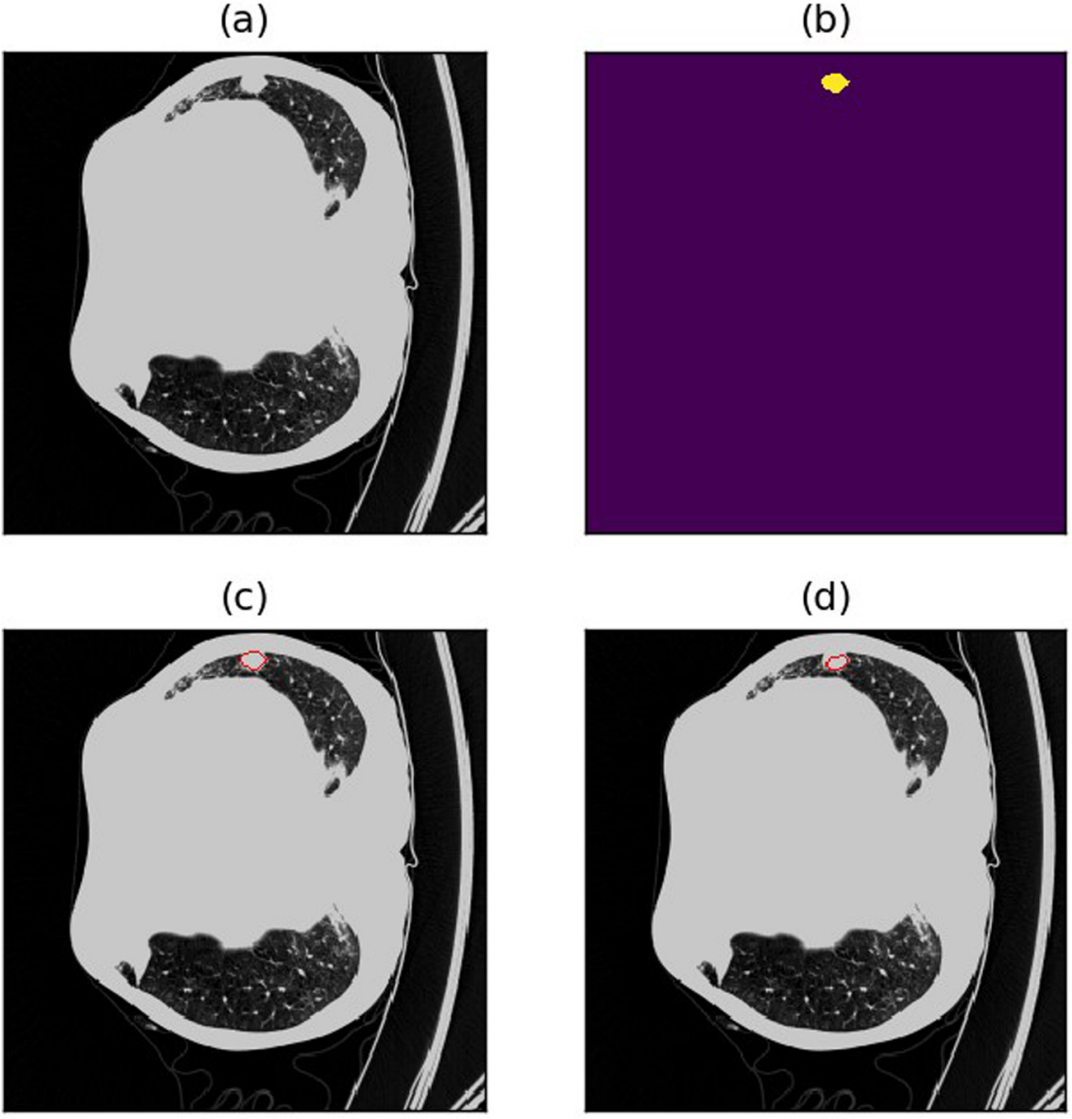
Example of poor detection outcomes due to lung nodules too close to the lung margins or other lung tissues. (a) Raw image. (b) Annotations (c) Annotated image. (d) Predicted image.

**Figure 13:**
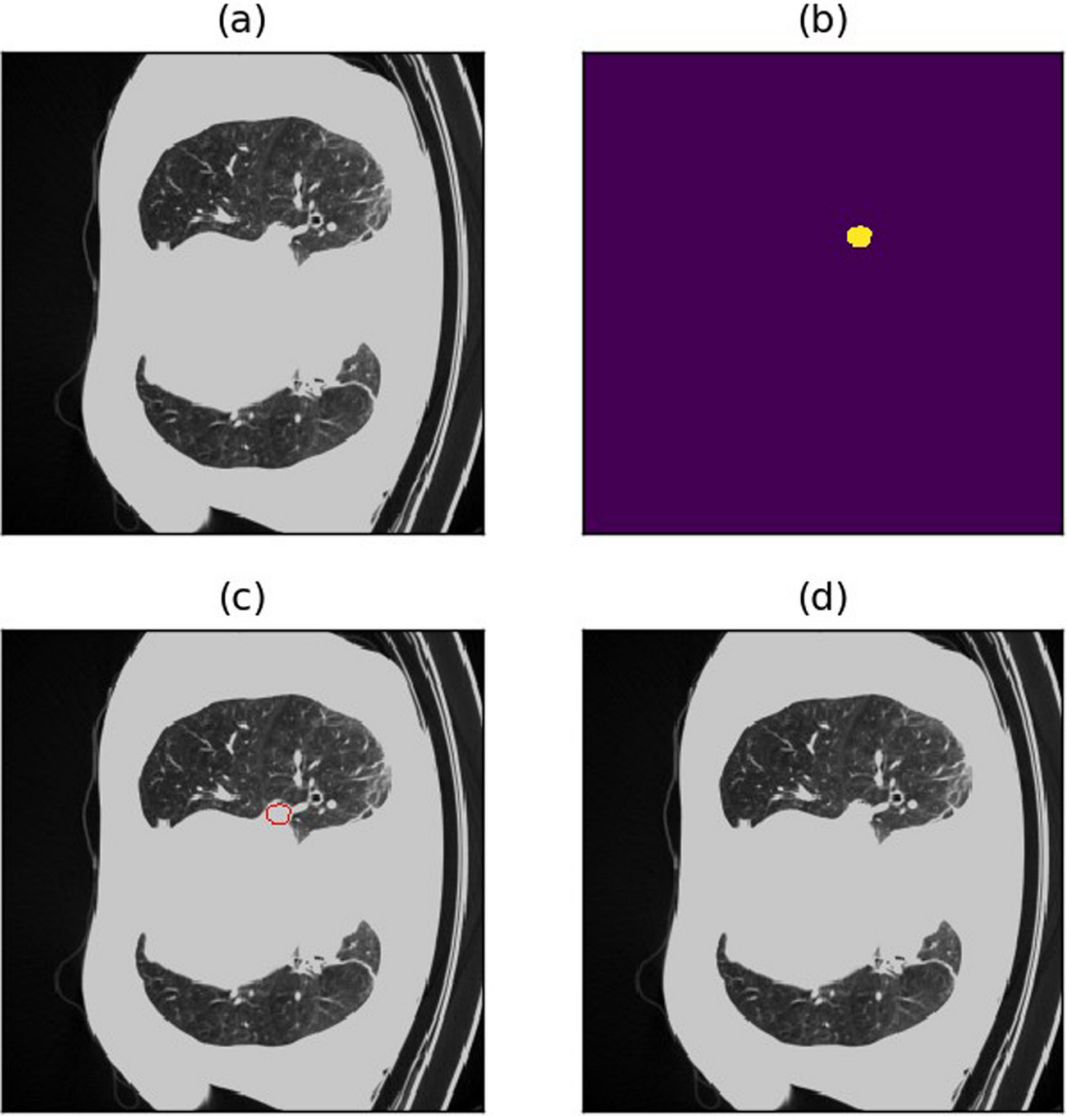
Example of missed detection due to lung nodules too close to the lung margins or other lung tissue. (a) Raw image. (b) Annotations (c) Annotated image. (d) Predicted image.

**Figure 14:**
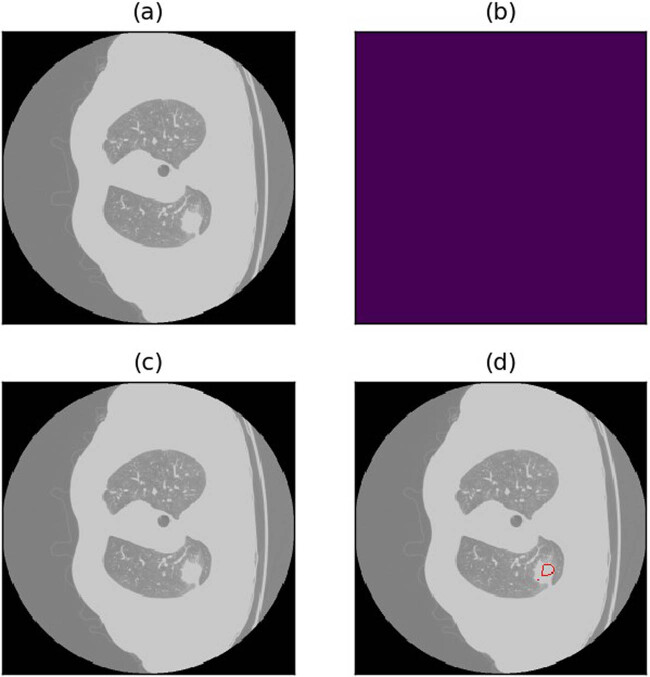
Example of misdetection due to the model’s inability to distinguish normal lung tissue from lung nodules. (a) Raw image. (b) Annotations (c) Annotated image. (d) Predicted image.

**Figure 15:**
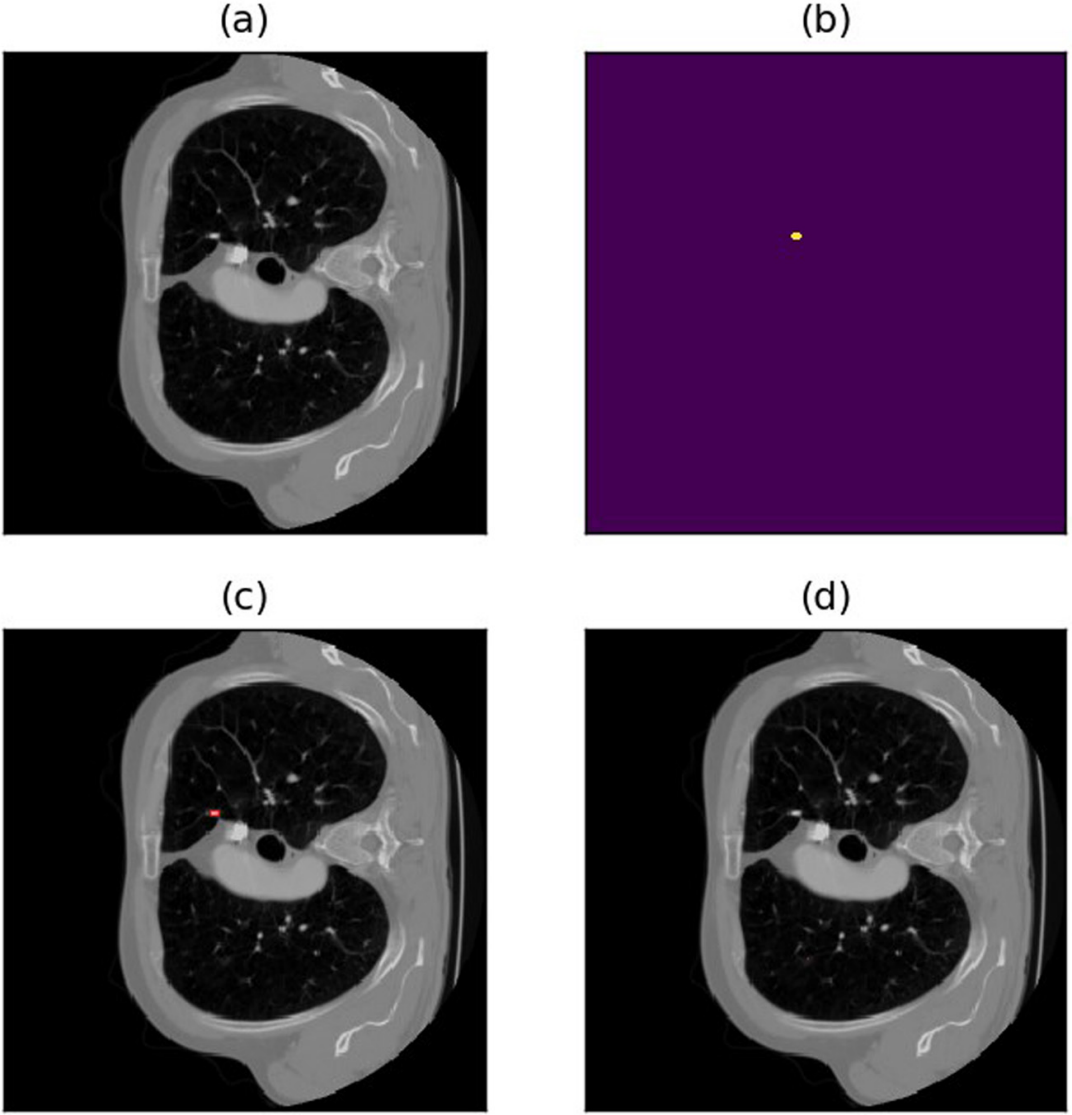
Example of missed detection of tiny nodules. (a) Raw image. (b) Annotations. (c) Annotated image. (d) Predicted image.

A dice loss of 0.4479 still falls short of meeting the accuracy threshold required for real-world applications. In this section, an analysis of potential scenarios where the model’s predictions fall short through visual observations was conducted. This examination offers insights that guide subsequent optimization efforts for the semi-residual MCNN. Several prevalent types of suboptimal cases include:

(1) Shrunk borders: The model predictions tend to be cautious, leading to predicted outcomes that are slightly smaller than the actual objects. This conservatism often results in a notable decrease in the dice loss, particularly for smaller nodules (refer to Figures 9 and 10 for illustrative examples).
(2) Missed detection due to lung nodules being in close proximity to the lung margins or other lung tissue. This challenge can arise for both large and smaller nodules (refer to Figures 11–13 for visual examples).
(3) Misdetection and missed detection instances emerge due to the model’s incapacity to differentiate between normal lung tissue and lung nodules (see Figure 14 for an illustrative example).
(4) Missed detection of diminutive nodules is prevalent. The likelihood of such missed detection rises notably when candidate nodules are exceedingly small (refer to Figure 15 for a visual example).

In conclusion, the model designed in this experiment generally performs well if the following conditions are satisfied:

(1) Candidate nodules have a moderate or large size.
(2) Candidate nodules are not too close to lung margins or other internal lung tissue.
(3) Image slice thickness is neither too small nor too large.
(4) The image noise level is low, and image boundaries could be clearly observed.

**Figure 16:**
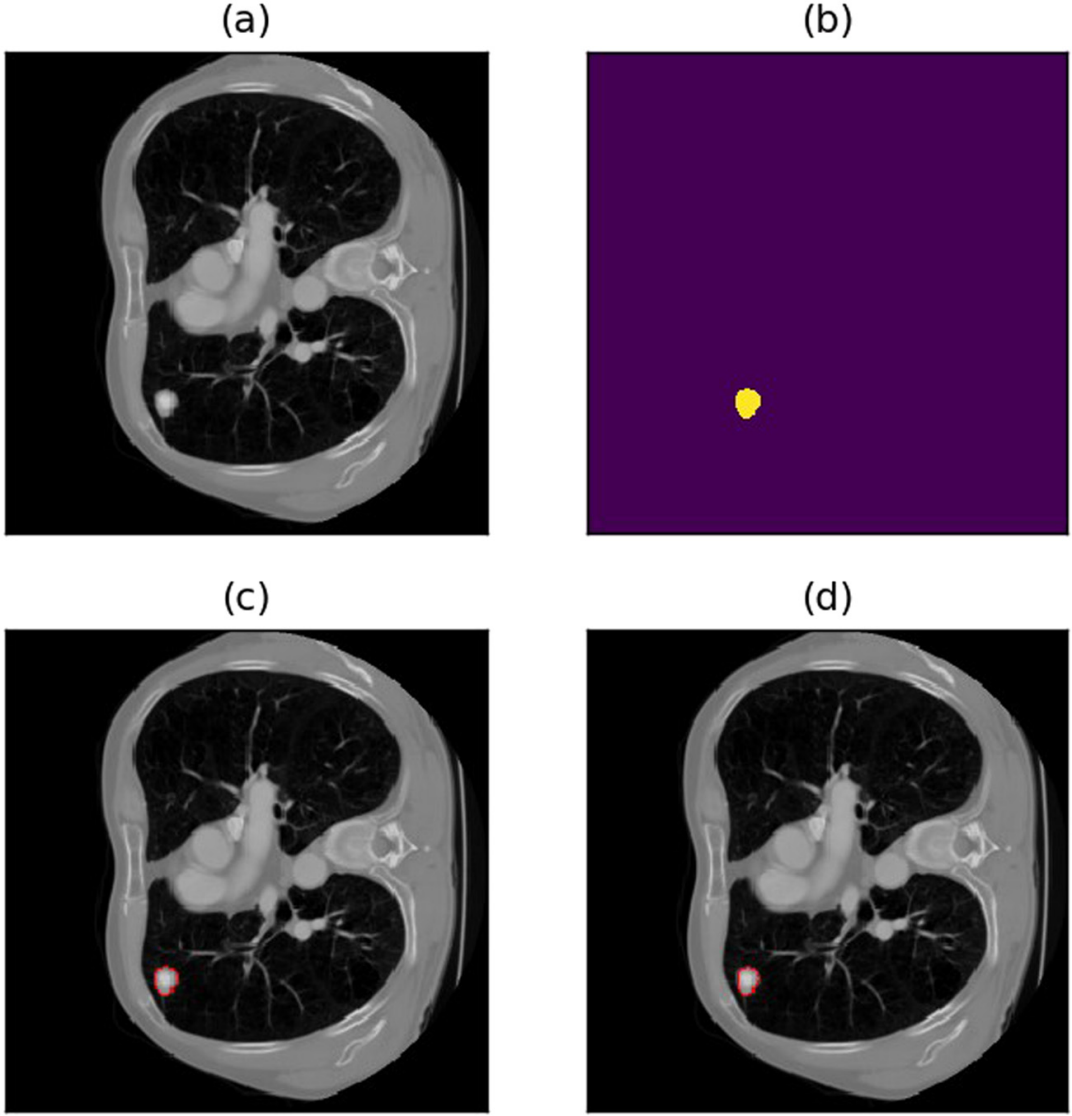
Example of a piece with good segmentation result. (a) Raw image. (b) Annotations (c) Annotated image. (d) Predicted image.

Figure 16 shows an example of good segmentation results, with a dice coefficient greater than 0.9.

## 4 Future work and conclusion

The primary objective of this study was to present an efficient approach for lung nodule segmentation utilizing lung CT images. To this end, a technique known as semi-residual MCNN for nodule segmentation was introduced. This method autonomously generates 3D nodule segmentation maps from lung CT images, minimizing the need for human intervention. The core innovations of the model encompass semi-residual building blocks, GN, and multi-resolution output heads. Each of these concepts has been demonstrated to hold pivotal significance in enhancing the overall accuracy of predictions.

Through this model, remarkable achievements have been realized, with dice coefficients reaching 0.4479 on the test subset of the LIDC-IDRI dataset. This notable performance surpasses that of a previously acclaimed state-of-the-art network, specifically the 3D Recurrent DenseNet, which exhibited dice coefficients of 0.4204 on the same dataset. The outcomes underscore the efficacy and advancements achieved by the proposed semi-residual MCNN technique in the realm of lung nodule segmentation.

The method proposed in this study, the semi-residual MCNN, serves as a notable advancement in enhancing the accuracy of neural networks for lung nodule segmentation tasks, building upon established methods. This progression not only paves the way for the integration of artificial intelligence into the clinical diagnosis of lung cancer but also offers valuable insights for real-life applications. Furthermore, the training methodologies and techniques outlined in this study exhibit a high level of adaptability and hold promising potential for application in analogous scenarios involving image segmentation tasks.

As underscored in preceding chapters, it is worth noting that the method proposed in this experiment does not delve into the specific delineation of individual nodules. Despite its notable improvements, the achieved accuracy still falls short of the requisites for real-world applications. Consequently, directing further efforts towards the exploration of two key domains is recommended:

(1) Data post-processing holds potential for significant enhancement. Introducing an additional module to refine the model’s prediction outcomes could furnish medical professionals with more actionable insights. This augmentation could encompass several valuable aspects, such as extracting pertinent information about each nodule’s size and shape, quantifying the total nodular count within an image, and even potentially evaluating the potential malignancy of individual nodules.
  By incorporating these refinements into the post-processing pipeline, the generated results could offer a comprehensive and nuanced view, equipping medical experts with invaluable details for informed decision-making. This augmentation not only complements the segmentation aspect of the model but also amplifies its utility in clinical practice.
(2) An alternative strategy involves the development of a two-stage detection system. Instead of directly generating a complete segmentation map, this approach entails a twofold process. In the initial stage, the model identifies and extracts the volumes of interest (VOI). Subsequently, in the second stage, precise segmentation maps are predicted for each extracted VOI [29].

This two-stage paradigm serves to mitigate certain challenges. Notably, it alleviates the model’s struggle in detecting smaller targets by focusing on localized VOIs. Moreover, it circumvents the predicament of imbalance between positive and negative samples in lung CT raw images. Consequently, this method holds the potential to elevate the efficiency and accuracy of detection. By strategically segmenting the task into two stages, this approach offers a refined and potentially more effective framework for lung nodule detection.
